# Bacillus Calmette-Guérin in the management of superficial bladder cancer

**DOI:** 10.4103/0970-1591.38608

**Published:** 2008

**Authors:** Rakesh Kapoor, Vivek Vijjan, Pratipal Singh

**Affiliations:** Department of Urology and Renal Transplantation, Sanjay Gandhi Post Graduate Institute of Medical Sciences, Lucknow, Uttar Pradesh, India

**Keywords:** Bacillus Calmette-Guérin, bladder neoplasm, immunotherapy

## Abstract

Intravesical Bacillus Calmette-Guérin (BCG) is the mainstay of superficial bladder cancer treatment. We performed a literature search through Medline/Pubmed using key words ‘Bacillus Calmette-Guérin’, ‘intravesical’, ‘bladder neoplasm’ and ‘immunotherapy’ for published data in the English language from 1970 to 2007 to review the current status of intravesical therapy and practice recommendations. The exact mechanism of action of intravesical BCG is yet to be elucidated. However, it appears that it is mediated by the local immune response, mainly through T-helper cell response. It reduces the recurrence rate by an average of 40% and progression by more than 20% in papillary tumors over the patients without BCG therapy. However, progression prevention is seen only in series which have used maintenance therapy at least for one year. It is effective in CIS of bladder with a response rate of more than 40% and prevention of progression in one-fourth patients. Most acceptable dose, induction treatment and maintenance therapy protocols are discussed. However, these are yet to be confirmed in large randomized trials. Intravesical BCG is well tolerated in most of the patients with mild to moderate side-effects in induction therapy; however, most patients do not complete maintenance therapy due to side-effects which is the most common concern at the present time.

Morales, Eidinger and Bruce were the first to report successful treatment of superficial bladder cancer with bacilli Calmette-Guerin (BCG) in 1976.[1] Since then, BCG has become the treatment of choice for high-risk superficial bladder cancer (SBC) in most countries of the world. Intravesical BCG therapy is regarded as the most successful immunotherapy to date.[2] It is not only superior to intravesical chemotherapy with regard to the recurrence rate of SBC[3,4] but also acts beneficially on the progression rate of this tumor.[5,6]

## MECHANISM OF ACTION OF INTRAVESICAL BCG

Bacillus Calmette-Guérin is an attenuated mycobacterium developed from the Mycobacterium bovis strain. The precise mechanism of action has not been clearly delineated; however, the pronounced infiltration of the bladder wall by immunocompetent cells together with the secretion of cytokines into the urine point toward the intense local immune activation after BCG.

As an initial step BCG contacts tumor cells through a novel fibronectin attachment protein which is followed by internalization of the BCG in the cells leading to the direct stimulation of cell-mediated immunologic response. A recent review has shown that it is predominantly a T-helper/inducer cell-mediated response with persistence of inflammatory (Th1-type) cytokines for a long time within the so-called BCG-induced granulomas, which might have an important role in the recurrence-free status of the patient.[7] This prolonged inflammation seems to provide immature effector cells with a continuous level of activating cytokines (such as IL-2, Interferon-γ and IL-12). *In vitro*, at least two cellular cytotoxic effector mechanisms have been determined, the well-known leukocyte-activated killer (LAK) cell cytotoxicity and a cytotoxic phenomenon termed as "the BCG-activated killer (BAK) cell phenomenon." The effector cells involved are activated NK cells, which are known to selectively kill malignant targets.[7]

Multiple cytokines can also be detected in the serum of BCG-treated patients, indicating that there is some degree of systemic response.[8]

## BCG VERSUS CHEMOTHERAPY: WHICH IS BETTER?

Many studies on patients with superficial transitional cell cancer (TCC) of bladder have shown a lower recurrence rate with BCG as compared to intravesical chemotherapy. A meta-analysis of six trials including 585 patients on BCG versus transurethral resection (TUR) of tumor alone showed that BCG provides significantly better prophylaxis of tumor recurrence in high-risk superficial TCC over TUR alone.[3] In terms of first recurrence, a 56% reduction in the hazard was attributable to BCG. The greatest benefit was observed in high-risk groups when at least one year of maintenance therapy was used.[9] The overall activity of BCG against papillary disease is twice that of chemotherapy in preventing recurrence.[4] Approximately 30% absolute advantage has been noted with BCG as compared to 15% absolute advantage with chemotherapy in comparison to TUR alone.[10] Similarly in CIS, BCG produces more than 70% complete response rate whereas the results of chemotherapy are usually around 50%.[9]

The current consensus is to employ intravesical chemotherapy in low-to-intermediate risk patients, whereas BCG remains the first-line treatment in high-risk patients as well as in patients with prior chemotherapy failures.[10]

## ROLE OF BCG IN RESIDUAL TUMOR

Bacillus Calmette-Guérin has an ability to eradicate residual small (<1.5 cm) tumors, however, it is associated with a higher recurrence rate than when it is given in prophylactic setting.[11] Several investigators have demonstrated a nearly 60% response by residual tumor with intravesical BCG alone.[12] However, it is mandatory that as much as possible a complete resection of tumor should be performed before embarking on intravesical BCG therapy.

## ROLE OF BCG IN RECURRENCE PREVENTION

It has been shown in multiple large series that intravesical BCG reduces the tumor recurrence after tumor resection by 20-65% for an average of about 40%. A recent systemic review of intravesical BCG plus TUR *vs.* TUR alone has shown that BCG provides 56% reduction in risk of first recurrence.[3] It has been found effective in decreasing the recurrence rate of high-grade lamina-invasive bladder cancers (T1G3) in many contemporary series.[13,14] In a series of 51 patients with T1G3 bladder tumor, Hurle *et al.* reported 25% recurrence rate at median follow-up of 85 months[13] while another series of 44 similar patients reported a recurrence rate of 27% at median follow-up of 28 months.[14] A systematic analysis of more than 30 randomized trials by the American Urological Association (AUA) bladder cancer clinical guideline panel reported a 30% decrease in recurrence rate by intravesical BCG therapy with tumor resection than tumor resection alone.[15]

## ROLE OF BCG IN CANCER PROGRESSION

It has been proved that BCG decreases the progression of bladder cancer. In a series of 403 patients with CIS, BCG reduced the risk of progression by 35% compared with intravesical chemotherapy.[13] In a randomized trial of 86 patients with high-risk SBC, Herr *et al.*,[16] demonstrated a greater delay in interval progression for BCG patients versus TUR controls. Additionally, the cystectomy rate was significantly decreased for carcinoma *in situ* patients treated with BCG (11% *vs.* 55% for TUR controls), as was the time to cystectomy.

Recently published two meta-analyses have addressed the issue of progression in detail. A meta-analysis by Sylvester *et al.*,[5] which included 24 trials, 2658 patients on BCG and 2205 patients without BCG, showed that the rate of progression was 9.8% for the BCG group *vs.* 13.8% for the non-BCG group with a 27% reduction in relative risk of progression (OR 0.73, *P* = 0.001) at a median follow-up of 2.5 years. The size of the treatment effect was similar in patients with papillary tumors and in those with CIS and there was no statistically significant difference in treatment effect for either overall survival or death due to bladder cancer.

In another meta-analysis of nine trials including 1277 patients of BCG and 1133 patients of Mitomycin C comparing the efficacy of intravesical BCG and Mitomycin C on tumor progression of superficial bladder cancer, Bohle and Bock[6] showed that relative risk of progression was reduced by 23% with BCG therapy (OR = 0.77, *P* = 0.08) at a median follow-up of 26 months which was not statistically significant. However, in the subgroup with BCG maintenance there was statistically significant superiority of BCG over Mitomycin C (OR = 0.66, *P* = 0.02).[6]

Both these trials showed risk reduction in progression of disease only on maintenance BCG therapy. Therefore, it was concluded in both the meta-analyses that maintenance therapy, at least for one year is required to achieve reduction in cancer progression.

## ROLE OF BCG IN CARCINOMA *IN SITU*

Intravesical BCG is the first-line therapy for carcinoma in situ (CIS) of the urinary bladder. In a meta-analysis of nine randomized trials including 700 patients with CIS, BCG therapy showed a complete response rate of 68% compared to 52% complete response in chemotherapy treated patients (*P* = 0.0002).[17] With a mean 3.6 years' follow-up, 47% BCG-treated patients showed no evidence of disease compared to 26% of chemotherapy patients. Patients treated with BCG also showed 26% reduction in disease progression. Persistent disease after a complete course of therapy is cause for concern. Herr and colleagues,[16] in a study of 180 patients, reported that progression at five years was 19% in initial responders but 95% in non-responders. The AUA guidelines panel supported BCG as the preferred initial treatment for CIS.[15]

## ROLE OF MAINTENANCE BCG

The Southwest Oncology Group (SWOG) reported the most significant impact of maintenance BCG therapy.[18] Patients received a six-week induction course followed by three instillations done weekly, at three and six months and every six months thereafter for three years. The estimated median recurrence-free survival was 76.8 months in the maintenance arm and 35.7 months in the non-maintenance arm (*P* < 0.0001). Average recurrence-free survival was 111.5 months in the non-maintenance arm and not estimable in the maintenance arm (*P* = 0.04). Overall five-year survival was 78% in the non-maintenance arm compared with 83% in the maintenance arm. No toxicities above Grade 3 were observed; however, only 16% of patients could tolerate the full-dose-schedule regimen and required reduction of the three booster treatment. Two-thirds of the patients who stopped BCG due to side-effects did so in the first six months suggesting that the side-effects did not increase appreciably with additional time on therapy. Moreover, the treatment group fared better despite most patients failing to complete the full maintenance schedule, which suggests that maximum benefit may have been achieved earlier and shorter maintenance schedules may accomplish the same result with less toxicity.

A meta-analysis of 24 randomized trials[5] showed that BCG significantly reduced the risk of progression after TUR in patients with intermediate and high-risk papillary tumors and those with CIS who receive maintenance BCG treatment. Bacillus Calmette-Guérin was only effective in trials with maintenance where it reduced the risk of progression by 37% (*P* = 0.00004). The reduction in the odds of death was not statistically significant at a mean follow-up of 2.5 years. Most authors believe that at least one year of maintenance therapy is appropriate. The optimum treatment schedule, whether monthly or as described in SWOG trial, remains unclear.

## OPTIMAL BCG DOSING

The goal of BCG therapy is to minimize the tumor burden by juxtaposing BCG and tumor cells. The optimal treatment schedule for BCG has yet not been established. Several studies suggest that a six-week induction course alone is insufficient to obtain an optimal response in many patients.[5,6] It is important to use sufficient rather than excess BCG. Excess BCG, in the form of repeated BCG cycles, on the contrary, suppresses immune response. Immune stimulation peaks at six weeks with initial induction course, but with subsequent courses it usually peaks at three weeks and the stimulation wanes with time.[19]

The average additional response to a second induction course is 25% in those treated for prophylaxis and 30% in CIS patients.[20] However, additional courses of BCG to treat refractory patients are accompanied by a significant risk of tumor progression (invasion or metastasis) in 20-50% of patients.[21] There is roughly a 7% actuarial risk of progression with every additional course of BCG therapy.[22] Therefore, patients demonstrating failure after one to two courses of intravesical therapy should be considered for alternative, more aggressive therapy. Response to BCG at six months can be used as a predictor of prognosis, with the number of patients developing progressive disease being significantly higher among nonresponders.[23]

## CURRENT STATUS OF LOW-DOSE BCG

Optimum dose of BCG remains to be defined. The potential for BCG dose reduction has been evaluated by several investigators. Martinez-Pinerio *et al.* have used standard 81 mg and reduced 27 mg dose of Connought strain of BCG in superficial bladder tumors.[24] The rates of recurrence (28% *vs.* 31%) and progression (11.5% *vs.* 13.3%) were similar in the two groups at a median follow-up of 69 months. However, significantly fewer patients had toxicity in the reduced dose arm resulting in delayed instillations or withdrawal. In a literature review by Cheng *et al.*, four of the five studies comparing low and standard doses of BCG showed a statistically significant lower toxicity.[25] In an EORTC Phase 2 trial, low and intermediate risk patients (Ta, T1, G1-2) were studied for the efficacy of reduced dose BCG.[26] A similar efficacy with a 61% complete response was noted with a quarter of standard dose.

In a recent study at our institute, 106 patients were randomized to three different doses of BCG (40 mg, 80 mg and 120 mg).[27] At a median follow-up of three years the Kaplan - Meier analysis for time to recurrence (*P* = 0.1839) and time to progression (*P* = 0.595) was not significantly different in the three treatment arms.

In general, the results seem to be better with standard dose BCG in multifocal and high-grade tumors with low dose regimens being restricted to patients with lower risk disease.[24]

Currently it is recommended to start intravesical BCG at least after 7-14 days of tumor resection or until postoperative bleeding is over.[10] No particular commercial strain or preparation has shown clinical superiority. It is administered one vial per 50 cc concentration under gravity with usual dwell time of two hours. Methods to decrease early voiding like eliminating residual volume, temporary abstinence from caffeine and overnight fasting may be worthwhile.[10]

## PREDICTING RESPONSE TO BCG

Proliferation rate, assessed by Ki-67 protein, has been shown to be a predictive marker of response to BCG in T1G3 tumors. Patients with <20% response were associated with a positive response to BCG installations.[28]

In a study at our institute, interleukin-8 (IL-8) was shown as a predictor for the response to standard (120 mg) and low (40 mg) dose intravesical BCG in superficial bladder cancer.[29] The IL-8 secretion after the initial intravesical BCG instillation strongly correlated with the possibility of future recurrence or progression.[29]

## MANAGEMENT OF BCG FAILURE

Patients failing to respond to first course of BCG can be considered for a second course of intravesical BCG and a response rate of 30-50% has been seen in this subgroup of patients.[12] However, patients who progress despite BCG, patients with multifocal T1G3 tumors and tumors associated with CIS may be a case for immediate cystectomy.[30]

Salvage regimens using interferon alone and combined with BCG have been employed in such situations. A complete response rate of 15-20% at one year has been seen with interferon-α alone.[31] Low-dose BCG combined with interferon-α (50-100 MU), has shown a one to two year success rate of 50-60%. A multi-institutional study showed a response rate of 42% at a median follow-up of 24 months.[32] Intravesical gemcitabine and taxanes and device-assisted intravesical therapies have been employed in BCG failures but remain largely investigational.

## ADVERSE EFFECTS OF INTRAVESICAL BCG

Most patients experience dysuria and urinary urgency and frequency that last for several days and worsen during the course of therapy. These side-effects may be treated symptomatically with mild anticholinergics, acetaminophen or phenazopyridine.

Most of the adverse effects occur during the first six months of therapy. In a study by the European Organization for Research and Treatment of Cancer (EORTC) 20% of the patients planned for 36 months of BCG therapy, stopped treatment due to local or systemic adverse effects.[33] Local toxicity remained constant during the maintenance therapy whereas systemic toxicity was predominantly seen in the first six months.

In the study conducted at our center adverse effects were seen in 55.6% patients with most being of Class 1 severity.[27] Significantly less patients developed severe adverse effects in the low-dose group as compared to the higher dose groups. The most common local reaction was cystitis and the commonest systemic adverse effect was fever and myalgia. No Class 4 toxicity was observed in any group.

## CONCLUSION

Intravesical BCG is the most effective therapy for CIS and superficial cancer of bladder. It provides a superior protection from tumor recurrence and even reduces disease progression. Despite its efficacy, BCG therapy is associated with frequent local and systemic adverse effects. Dose reduction of BCG has been associated with a corresponding reduction in BCG-related toxicity. However, at present most authorities recommend standard dose (80 mg) therapy. Six-week induction therapy is standard of care to prevent recurrence; however, maintenance with BCG immunotherapy for at least one year is advocated to prevent tumor progression. The goals of reducing side-effects and improving the tolerance of BCG should form the basis of an ideal maintenance schedule.
